# Determination of loyalty among high school students to retain in the same university for higher education: An integration of Self-Determination Theory and Extended Theory of Planned Behavior

**DOI:** 10.1371/journal.pone.0286185

**Published:** 2023-11-08

**Authors:** Ardvin Kester S. Ong, Yogi Tri Prasetyo, Venice Cristine C. Dangaran, Mark Anthony D. Gudez, Julius Ivan M. Juanier, Gabriel Andrey D. Paulite, Rohn Xavier R. Yambot, Satria Fadil Persada, Reny Nadlifatin, Irene Dyah Ayuwati

**Affiliations:** 1 School of Industrial Engineering and Engineering Management, Mapúa University, Manila, Philippines; 2 E.T. Yuchengco School of Business, Mapúa University, Manila, Philippines; 3 International Bachelor Program in Engineering, Yuan Ze University, Chung-Li, Taiwan; 4 Department of Industrial Engineering and Management, Yuan Ze University, Chung-Li, Taiwan; 5 Malayan High School of Sciences, Maynila, Philippines; 6 Young Innovators Research Center, Mapúa University, Manila, Philippines; 7 Entrepreneurship Department, BINUS Business School Undergraduate Program, Bina Nusantara University, Jakarta, Indonesia; 8 Department of Information Systems, Institut Teknologi Sepuluh Nopember, Surabaya, Indonesia; 9 Department of Information Systems, Institut Teknologi Telkom Surabaya, Surabaya, Indonesia; Shanghai Ocean University, CHINA

## Abstract

Student loyalty generally refers to the formed bond between the student and a university. This relationship between a university and its students proves essential in a university’s success in the competitive field of higher education institutions. The aim of this study was to determine the factors affecting students’ loyalty among high school students to pursue their college or higher education in their current universities by utilizing Self-Determination Theory and Extended Theory of Planned Behavior. A total of 1224 high school students voluntarily participated and answered an online questionnaire that consist of 80 questions. Structural Equation Modeling (SEM) showed that competency had the highest direct significant effect on perceived behavioral control which subsequently led to student satisfaction, followed by relatedness and empathy. In addition, student satisfaction had the highest direct effect significant effect on student loyalty, followed by university image and effectiveness. Interestingly, university ranking, programs offered, and kinship patronage also had significant indirect effects on student loyalty. This new framework may be a theoretical foundation for universities to enhance student loyalty and student recruitment. Considering students as customers, the satisfaction of students would result in an increase in the application which would present an increase in population, sales, marketability, and profitability of the university.

## 1. Introduction

Student loyalty generally refers to the formed bond between the student and an institution. This bond reflects the sense of belongingness felt by the students toward their university [1]. Due to the established relationship, student loyalty may result in various forms of support in institutional endeavors such as the intention to continue their studies. Likewise, student loyalty extends to former students of an institution and is exhibited in terms of providing financial assistance to a university. This relationship between an institution and its students proves essential to a university’s success in the competitive field of higher education institutions.

Contemporary studies demonstrate the importance of student loyalty in higher education institutions. Jones et al. [2] stated that loyal students tend to have a firm commitment to their institutions, resulting in retention despite presentable options and opportunities. Mesta [3] described student loyalty as a critical aspect in navigating competition between universities that interprets loyalty as the foundation of positive word-of-mouth that attracts prospective students. Loyalty reflected from other studies [4–7] is defined as the students’ pursuance and objective feeling when it comes to choosing their current institution for higher education, choosing the institution despite available choices, and talking to others to consider the current institution. Thus, insight into student loyalty is essential to university development and stability. This research situates itself in the increasing field of student loyalty studies, specializing in determining high school students intentions in pursuing collegiate studies at their current university.

The trends in focus on student loyalty have proven essential in various parts of the world. In Asia, higher education universities are challenged in recruiting more students amid the opportunities for senior high school students [8]. Countries in Europe also conducted studies regarding the management of student loyalty to benefit from the modern competitive market [9]. In the global field, international students choose universities by evaluating course programs and locations amplifying the competition between universities. These obstacles resulted in various efforts of universities around the world in addressing student loyalty, retention, and recruitment, such as treating students as customers of higher education universities.

The efforts conducted by universities in understanding student loyalty allow the determination of its antecedents. Chandra et al. [10] discussed that service quality, university image, and student satisfaction were used as latent variables. Likewise, Giner and Rillo [4] stated that satisfaction was used as one of the main factors of loyalty. Although several studies have demonstrated the application of student loyalty [10, 11], most were only focused on either college students or prospective students of a university. A significant gap can be drawn in the field of student loyalty among high school students, particularly their intention to stay in the same university for college or higher education. This intention of pursuing higher education in their current university can be discussed using different theories such as the Self-Determination Theory (SDT) and the Theory of Planned Behavior (TPB) [12–14].

Although multiple studies have incorporated SDT and TPB in the educational sector [15], there are limited discussion and identified factors affecting the intentions of high school students to continue their higher education in the same university. Al-Jubari et al. [16] conducted a study on the entrepreneurial intention of university students in Malaysia. The study confirmed the positive effects of intrinsic and extrinsic motivation towards intention. However, the researchers suggested that both motivations vary from person to person. This resulted in differences in intention, particularly satisfaction and frustration. Chandra et al. [10] proposed several factors in determining the student loyalty of Indonesian university students. The research considered satisfaction, service quality, and image as the primary antecedent of loyalty. Results showed a significant and direct relationship between satisfaction and image to loyalty, while service quality revealed no significant correlation with loyalty. A study of Chilean private school students by Gallegos and Vasquez [11] demonstrated the relational effect of commitment, trust, and satisfaction on student loyalty. The researchers discussed the linear formation of loyalty that develops from satisfaction, then continues to trust and commitment that culminates in loyalty. Despite the comprehensive description of loyalty formation, the study only focused on the affective factors of loyalty and did not acknowledge the implications of physical or procedural attributes of a university. Therefore, this paper argues that a complete understanding of loyalty can be formed through the lenses of satisfaction as the behavioral attribute and university image as the physical characteristics of a university, both affecting student loyalty.

A reoccurring theme is evident among the discussed studies involving SDT, TPB, satisfaction, and university image. It could be seen that there is limited research and understanding of factors affecting high school student retention and loyalty in pursuing higher education in the same university. This gap instigated the aim of this study in determining factors affecting students’ loyalty among high school students to pursue higher education in their current institutions. Consequently, the created framework of this study could be used in determining student loyalty to secondary and tertiary institutions. The relationships between the variables in this research may also be applied in fields beyond education, where customer loyalty is the primary focus. Considering students as customers, the satisfaction of students would result in herd application. The increase in the application would result in an increase in population, sales, marketability, and profitability for the university.

## 2. Theoretical research framework

Presented in Table 1 are the built hypotheses which are described in the succeeding section. There were 15 hypotheses built from the integrated framework of SDT and extended TPB to measure student loyalty with university image and satisfaction as its primary variables. SDT is a theory specializing in human motivation, intentions, and general behavior. This framework has been used in measuring students’ intrinsic motives such as autonomy, competency, and relatedness [13, 17]. Several studies have incorporated SDT in the educational field. Hobson and Maxwell [18] utilized SDT to evaluate the well-being of early secondary school teachers. The results supported the interconnectedness of the three latent variables. Moreover, Kaur et al. [19] used SDT to determine student drop-out intentions. The study found the significant effects of autonomy, perceived motivation, and perceived competency on the drop-out intention of students. Similar to the other studies, the research mentioned the importance of the three dimensions of SDT and implied that the fulfilment of the three psychological needs results in greater motivation among students. In further determining human behavioral intentions, Webb et al. [12] integrated SDT with TPB.

**Table 1:** Hypotheses from integrated framework.

Hypothesis Number	Claim
**1**	Tuition fee has a significant direct effect on university image.
**2**	The admission process has a significant direct effect on university image.
**3**	The programs offered have a significant direct effect on university image.
**4**	School facility has a significant direct effect on university image.
**5**	Faculty profile has a significant direct effect on university image.
**6**	Kinship patronage has a significant direct effect on university image.
**7**	University ranking has a significant direct effect on university image.
**8**	University image has a significant direct effect on student loyalty.
**9**	Affective has a significant direct effect on student loyalty.
**10**	Autonomy has a significant direct effect on university image.
**11**	Relatedness has a significant direct effect on university image.
**12**	Competency has a significant direct effect on university image.
**13**	Empathy has a significant effect on perceived behavioral control.
**14**	Perceived behavioral control has a significant effect on student satisfaction.
**15**	Satisfaction has a significant effect on student loyalty.

The TPB is a theory used to predict human behavior. TPB specializes in determining the intention to perform a specific action [14, 20, 21]. TPB describes human behavior as the outcome of a rational thought process. Under TPB, the overall intention to pursue a goal is predicted by the different latent constructs of attitudes, subjective norms, and perceived behavioral control [22]. Educational sector applications of TPB, such as pro-environmental behavior of high school students [23], the adaptation of mobile devices to courses [24], and mobile distance learning [25], provide insight on intentions of students. These distinct behavioral characteristics are measured primarily through perceived behavioral control, which includes both feeling and perception of controllability of an action [16]. From the description and applications of the theory, TPB proves applicable in the field of student intention of pursuing higher education in the same university [26].

Economic values, especially TF, are factors that consequently affect the overall development of UI. Similarly, admission or entry requirements affect university image formation [27, 28]. In the study of Palacio et al. [29], one of the factors that affected UI was the ease of university entrance. In addition, UI consists of the subjective viewpoints of students about the quality of the programs and the university’s social and physical environment [30]. The PO are evaluated factors of UI assessed by students, and it determines the overall value within the market [26]. Therefore, the following were hypothesized:

**H1**. Tuition fee has a significant direct effect on university image.

**H2**. The admission process has a significant direct effect on university image.

**H3**. The programs offered have a significant direct effect on university image.

SF and the physical environment of an institution generally affect UI [27]. According to Luque-Martinez and Del Barrio-Garcis [31], SF such as furnishing, physical space, computer equipment, and other technological facilities influence the UI. Thus, the measurement of school facilities affects student satisfaction with university’s image. Several studies also included FP as a determiner of an institution’s image, defining FP as the summation of attitudes and behavior of those in charge of the university [30, 32]. Therefore, the following were hypothesized:

**H4**. School facility has a significant direct effect on university image.

**H5.** Faculty profile has a significant direct effect on university image.

Loyal alumni can support the university through finances or recommendations to future students since any kind of positive word of mouth about a university significantly affects its general image [33]. Recommendations by the alumni of an institution impact the profitability and overall success of the university [1]. Studies showed that most students follow or consider their parental advice when choosing a university [34]. Thus, students favor their KP or their parents’ collegiate institutions when selecting a university. Aside from KP, UR positively affects the UI [28, 35]. Therefore, the following were hypothesized:

**H6.** Kinship patronage has a significant direct effect on university image.

**H7.** University ranking has a significant direct effect on university image.

According to Nguyen and LeBlanc [36], image influences customer loyalty. Applying in the context of higher education institutions, Wilkins and Huisman [37] demonstrated the influence of opinions gathered from personal relationships and media on the choice of institution and retention of students. Chandra et al. [10] determined that image positively and significantly impacts SS and SL. Therefore, this was hypothesized:

**H8.** University image has a significant direct effect on student loyalty.

Customer loyalty is the long-term relationship between the service provider and the service receiver. Since higher education is a form of service, its students act as the core customers [26]. Consequently, loyalty involves AF and CO traits [2]. AF traits describe emotions, whereas CO traits describe people’s judgment and thought processes [38]. Therefore, this was hypothesized:

**H9**. Affective has a significant direct effect on student loyalty.

The belief in carrying a behavior depends on whether people consider themselves to have sufficient resources and opportunities and when they feel liberty in making decisions to use presumed resources and opportunities [39]. Yzer [39] described perceived competency as the degree of students that enables their PBC. Sibthorp et al. [40] defined AU as a student’s belief in having control over their choices within the institution. Furthermore, both CO and AU constructs established PBC [22]. Therefore, the following were hypothesized:

**H10**. Autonomy has a significant direct effect on university image.

**H11**. Relatedness has a significant direct effect on university image.

**H12**. Competency has a significant direct effect on university image.

Service settings, such as higher education universities provide interactions with their customers. Such interactions may depict levels of courtesy through EM that may resonate with the receiver, resulting in changes in satisfaction and consumer intentions [41]. According to Aggarwal et al. [42], staff empathy towards its customers acts as a positive trait that can build long-term trust and satisfaction, leading to loyalty. Moreover, institutional empathy also provides insight into the specific part of human emotion that leads to motivation. Therefore, this was hypothesized:

**H13**. Empathy has a significant effect on perceived behavioral control.

PBC influences the satisfaction process, where is the most important in predicting a student’s future options [43]. Several studies also support the positive effect of perceived control on satisfaction [44, 45]. The relationship between SS and SL was evident in multiple studies [8, 46]. Consequently, Khadka and Maharjan [47] found that satisfied students are more loyal to their universities than those who are dissatisfied. Therefore, the following were hypothesized:

**H14**. Perceived behavioral control has a significant effect on student satisfaction.

**H15**. Satisfaction has a significant effect on student loyalty.

**Fig 1:**
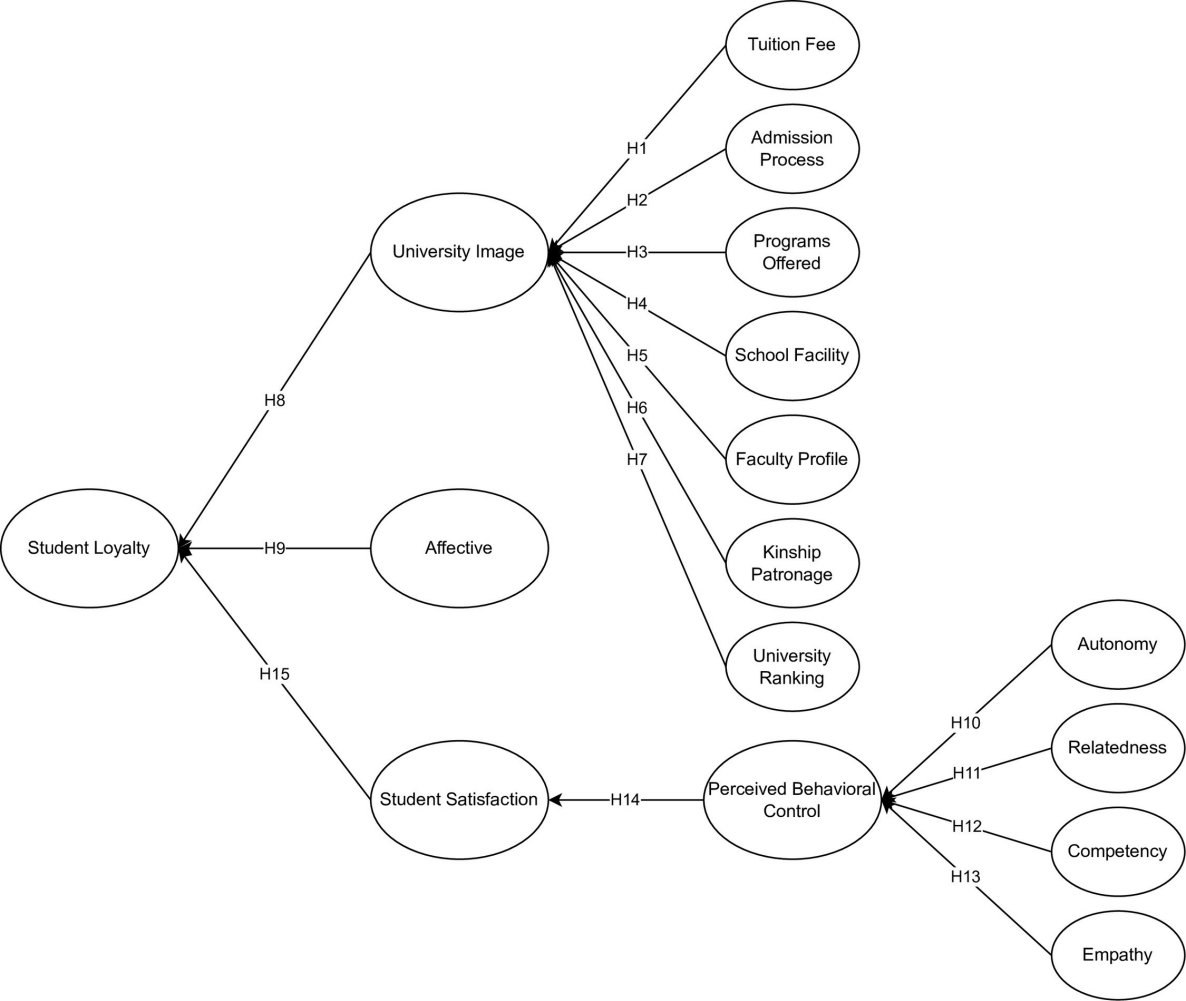


Fig 1 represents the theoretical research framework of the study. This study considered different latent for university images such as tuition fee (TF), admission process (AP), programs offered (PO), school facility (SF), faculty profile (FP), kinship patronage, and university ranking [48]. For satisfaction, this research considered the perceived behavioral control (PBC) under the TPB as latent [44, 45]. Lastly, autonomy (AU), competency (CO), relatedness (RE), and empathy (EM) were considered antecedents for the PBC latent [16, 49].

## 3. Methodology

This study was approved by Mapua University Research Ethics Committee. Each respondent was instructed to fill out a consent from which followed the Data Privacy Act or Republic Act No. 10173 in the Philippines. In addition, each respondent was also informed clearly about the purpose of the study prior to the data collection and was required to sign the consent form.

### 3.1. Participants

The questionnaires were disseminated virtually using Google forms. The distribution of the survey forms started from March 20 to March 29, 2021, using convenience sampling methods [50]. A total of 1224 high school students from Grades 9–12, aged between 14–20 years old responded to the online questionnaire voluntarily. All of the respondents that were collected came from the capital of the country and are studying in institutions from the capital, all of which are from private institution that offers higher education. Upon checking the respondents using SPSS 25, no missing data were seen. In addition, normality test using Shapiro-Wilks presented a quotient within the range of ±1.96 which indicates that the data is normally distributed [51]. In addition, the analysis using Harman’s Single Factor Test for Common Method Bias (CMB) was conducted. With threshold of 50%, the collected data presented a result of 25.16% which indicates no CMB [52].

Table 2 represents the descriptive statistics of the demographics among the respondents. Among the 1224 respondents, 50.7% were male and 48.8% were female. Majority of them come from ages 17 and 18 years old, 44.9% and 35.7% respectively. Most of the students were in Grade 11, 49.2% and Grade 12, 46.2%. Lastly, the monthly salary of more than 75,000 PHP with 7%, 13.5% with less than 15,000 PHP, 14.6% with 15,000–45,000 PHP, 16.1% with 30,000–45,000 PHP, 16.1% with 45,000–60,000 PHP, and 34.6% with 60,000–75,000 PHP were seen. The salary of parents according to the study by Basaluddin et al. [53] affects students’ higher education institutions. It was seen that when parents’ salary is in the lower bracket, then institutions with lower tuition fees will be more favorable. In this case, it was seen that most of the parents are capable to choose whichever university in the Philippines with a higher monthly salary.

**Table 2:** Descriptive statistics of the respondents (*N = 1224*).

Characteristics	Category	N	%
Gender	Male	620	50.7
	Female	597	48.8
	Other	7	0.6
Age	14	10	0.8
	15	34	2.8
	16	141	11.5
	17	549	44.9
	18	437	35.7
	19	48	3.9
	20	5	0.4
Grade Level	Grade 9	37	3.0
	Grade 10	19	1.6
	Grade 11	602	49.2
	Grade 12	566	46.2
Salary of Parents	< 15,000 PHP	86	7.0
	15,001–30,000 PHP	165	13.5
	30,001–45,000 PHP	179	14.6
	45,001–60,000 PHP	197	16.1
	60,001–75,000 PHP	174	14.2
	> 75,000 PHP	423	34.6

### 3.2. Questionnaire

Following the theoretical framework (Fig 1), constructs were adapted as seen in Table 3 to form a self-administered questionnaire to determine students’ university loyalty and its antecedents: institutional image and student satisfaction. The questionnaire consisted of eighteen sections: (1) Consent of the respondents (2) Demographic Profile of the respondents, (3) SL, (4) TF, (5) AP, (6) PO, (7) SF, (8) FP, (9) KP, (10) IR, (11) UI, (12) AF, (13) AU, (14) RE, (15) CO, (16) EM, (17) PBC, and (18) SS. A 5-point Likert scale was utilized to measure all the latent constructs measuring using the SEM. Prior to the distribution of questionnaire, a pre-test with the Ethical Committee of Mapua University assessed the questionnaire (FM-RC-21-75). In addition, a preliminary assessment for the overall acceptability of the questionnaire was administered with 100 respondents, showing a Cronbach’s alpha value greater than 0.70. Thus, the questionnaire was fully utilized and distributed.

**Table 3:** The constructs and measurement items.

Construct	Items	Measures	Supporting References
Student Loyalty	SL1	I intend to continue my college studies in the same institution.	Ginner and Rillo [4]
	SL2	I would recommend my current school to prospective students.	Snijders et al. [6]
	SL3	I would choose my present institution if I had to choose a school for college right now.	Helgesen and Nesset [5]; Paul and Pradhan [7]
	SL4	I often talk to my peers about the benefits of joining my current school.	Paul and Pradhan [7]
Tuition Fee	TF1	My family’s financial capacity plays a significant role in choosing my school for college.	Lindheimer [54]
	TF2	I consider tuitions fee when choosing schools.	
	TF3	I consider schools that offer financial discounts.	
	TF4	The tuition fee in my choice is reasonable.	
Admission Process	AP1	I consider the admission process of the school/s that I choose.	IvyWise [55]
	AP2	I consider schools that offer/s online admission.	
	AP3	The admission process of my current school is easy to understand.	
	AP4	My current school has reasonable admission requirements.	
Programs Offered	PO1	The programs of the institution offer practical and useful content that can be utilized for future use.	Teeroovengadum et al. [56]
	PO2	The college programs in my current school offer high-quality academic standards.	Teeroovengadum et al. [56]
	PO3	My institution offers programs with high quality education.	
	PO4	The programs offered by my current institution ensure student development.	
	PO5	My institution offers various courses to fit every student.	Wilkins and Huisman [28]
	PO6	My current institution offers the course that I want to take.	
School Facility	SF1	My school has overall quality facilities.	
	SF2	My school offers advanced technology or special learning equipment in their facilities.	
	SF3	My school is equipped with sufficient laboratories.	
	SF4	My school has adequate facilities for learning.	
	SF5	My school has sufficient on-site service facility such as clinics, school canteen, and counselling center.	
	SF6	My school has adequate recreational facilities.	
	SF7	My school has good classroom conditions.	
	SF8	My school offers clean and well-maintained comfort rooms.	
Faculty Profile	FP1	Professors are up to date with information regarding their field of profession.	Teeroovengadum et al. [57]
	FP2	The instructors are highly qualified in their field of teaching.	
	FP3	The instructors give individual attention to each student.	
	FP4	The instructors understand what each student needs.	
	FP5	I am satisfied with how the instructors deliver lectures.	
Kinship Patronage	KP1	My alumni relatives highly recommend my current institution.	
	KP2	My alumni relatives significantly affect my choice of school.	
	KP3	I chose my current institution because of my relatives are alumni of the school.	
	KP4	The alumni of my school highly recommend the institution.	
University Ranking	UR1	I consider institutional ranking when choosing a school.	
	UR2	My school has a great Institutional Ranking.	Teeroovengadum et al. [57]
	UR3	My school has a considerable student to academic staff ratio.	
	UR4	My school has a high academic reputation such as awards and highly cited researchers.	
	UR5	I feel that my school is greater than other schools.	
University Image	UI1	I choose schools based on their level of prestige.	Teeroovengadum et al. [57]
	UI2	I have heard that the graduates of my current institution have become successful.	
	UI3	I have always had a good impression of my current school.	Schlesinger et al. [58]
Affective	A1	I think my current institution is the best choice among my other school choices.	Orozco and Arroyo [59]
	A2	I am willing to make all efforts in helping my current school achieve its goals.	Orozco and Arroyo [59]
	A3	I feel proud to study in my current institution.	Annamdevula and Bellamkonda [60]
	A4	I feel a strong sense of belonging in my current school.	
	A5	I feel a strong sense of identification with my current school.	
Autonomy	AU1	My current campus provides activities that enable us to use our own set of skills.	
	AU2	The teachers in my current school provide me with choices and options.	
	AU3	I do not feel restricted when I’m at my current school.	
	AU4	Our professors encourage us to ask questions.	
	AU5	I feel that I can decide on my own freely.	
Relatedness	RE1	My relationship with my family, friends, classmates, and teachers influences my decision when choosing schools.	
	RE2	I feel the support of my family, friends, classmates, and teachers when choosing a school.	
	RE3	I feel the social support of the people in my current school.	
	RE4	I feel connected and part of my current school.	
	RE5	I feel that my friends will go to the same institution as me.	
	RE6	My friends encourage me to enroll in the institution where they are at.	
	RE7	My parents/relatives encourage me to enroll in the institution of my choice.	
Competency	CO1	I can set my preferences in choosing a school.	
	CO2	I apply my past and current experiences to set my preferences in choosing a school.	
	CO3	I can distinguish between the schools I want, and I do not want.	
	CO4	I can select the school I want based on my preference.	
	CO5	Overall, I believed that choosing a school based on my preferences will be beneficial to me.	
Empathy	EM1	My school makes an effort to understand students’ needs.	Izogo and Ogba [61]
	EM2	My current school knows the needs of its students.	
	EM3	I believe that my current school has reasonable schedules convenient to its students.	
	EM4	My school actively responds to students’ enquiries.	Izogo and Ogba [61]
	EM5	My school gives students individual attention to its students.	Izogo and Ogba [61]
Perceived Behavioral Control	PBC1	I am confident that I could stay in the same school if I want to.	Shin and Hancer [62]
	PBC2	I have the knowledge and control where to enroll.	
	PBC3	I think that I am capable of choosing a school for college on my own.	
	PBC4	I have the overall control in choosing a school for college.	
	PBC5	I will choose the institution I will be going to.	
Student Satisfaction	SS1	In general, I am satisfied with the services offered by my current school.	Ginner and Rillo [4]
	SS2	I am pleased with my decision to enroll on this campus.	
	SS3	I am satisfied to study on the same institution I am currently at.	
	SS4	My experience in my school has been enjoyable.	
	SS5	I am enjoying studying at my current school.	Teeroovengadum et al. [57]

### 3.3 Structural Equation Modelling

AMOS 26 and SPSS 25 were utilized in this study to calculate SEM. SEM is an advanced statistical tool that calculates the causal relationship among the different constructs in a framework [63]. SEM specializes in different disciplines such as behavioral and social sciences with its theory incorporation capability through quantitative measures [64]. Thus, this study utilized SEM in measuring high school student retention intentions in the same university for college or higher education by integrating the Extended TPB and SDT.

## 4. Results

**Fig 2:**
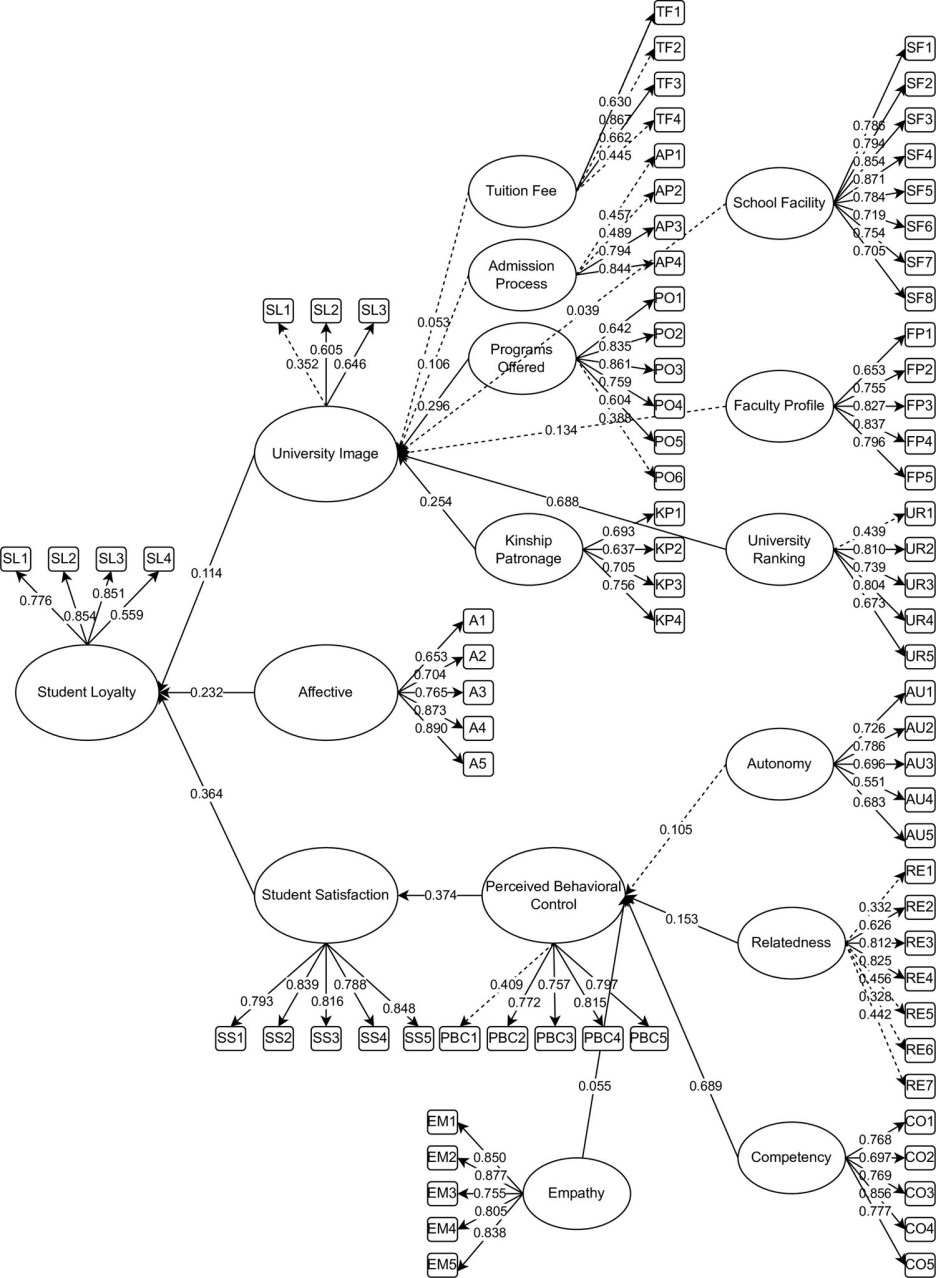
The initial SEM with indicators for determining the factors affecting high school students’ retention intentions in the same university for college.

**Fig 3:**
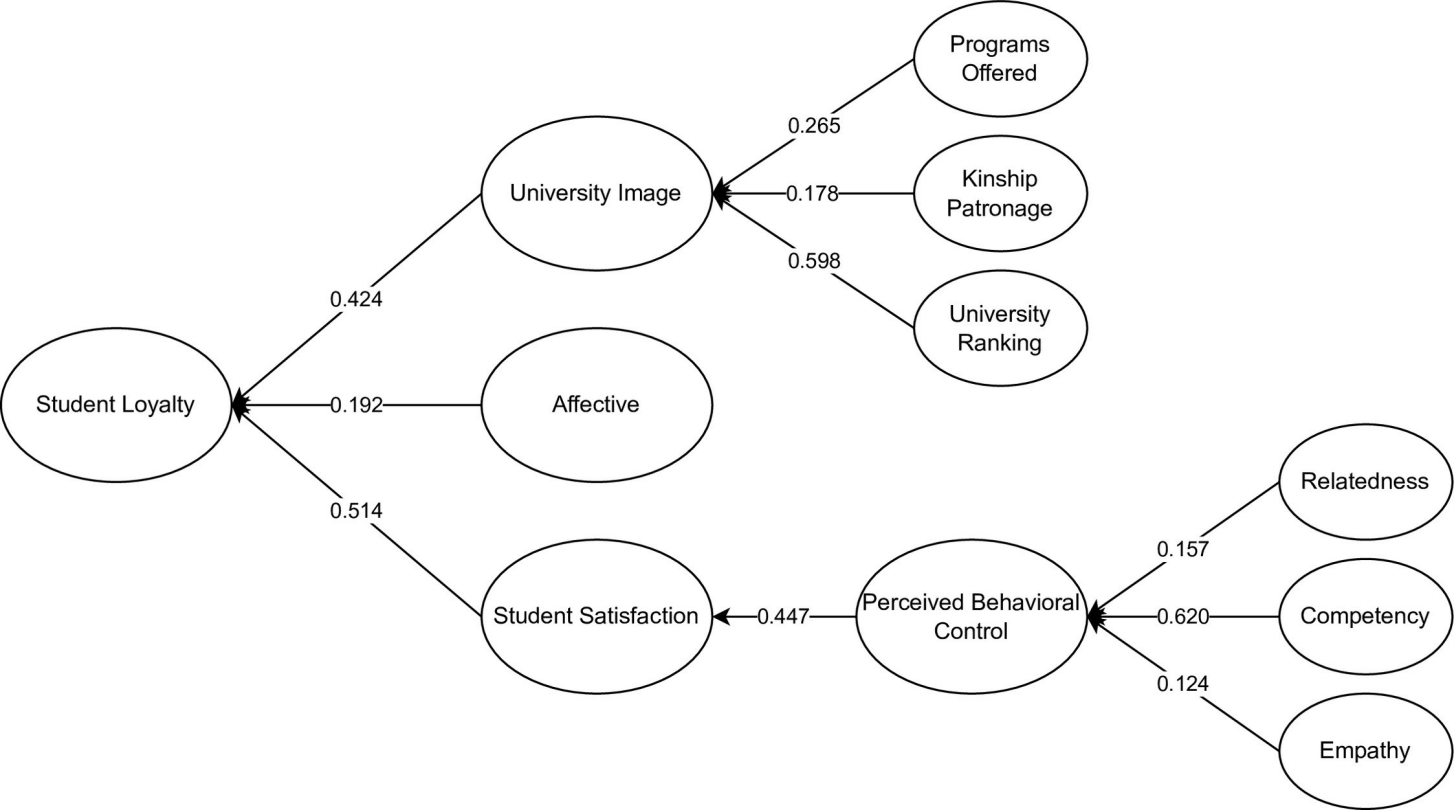
The final SEM for determining the factors affecting high school students’ retention intentions in the same university for college.

Fig 2 represents the initial SEM with indicators for determining the causal relationship between the latent variables affecting high school students’ retention intentions in the same university for college. It could be seen that TF, AP, SF, FP, and AU were not significant. Following the suggestion of Hair [63], removing the non-significant latent (p-value < 0.05) and constructs (< 0.5) could be done to enhance the model fit of this study. Fig 3 represents the final SEM with the significant latent constructs affecting high school students’ retention intentions for the same university for college.

Table 4 presents the descriptive statistics of the initial and final SEM factor loading for determining the factors affecting high school students’ retention intentions in the same university for college. In addition, Table 5 presents the composite reliability, which displays the validity among the different constructs together with their latent. The values of Cronbach’s alpha and Composite Reliability were above the minimum acceptable range of 0.700 [63]. Moreover, the values of the Average Shared Variance (AVE) were lower than the accepted value of 0.500 indicating consistency among the constructs [63].

**Table 4:** Indicators statistical analysis.

Variable	Item	Mean	StDev	Factor Loading	
				Initial	Final
Student Loyalty	SL1	3.011	1.277	0.776	0.733
	SL2	3.819	0.959	0.584	0.677
	SL3	3.230	1.243	0.851	0.851
	SL4	3.315	1.416	0.559	0.580
Tuition Fee	TF1	4.003	1.096	0.630	-
	TF2	4.086	1.065	0.867	-
	TF3	4.068	1.050	0.662	-
	TF4	3.802	0.941	0.455	-
Admission Process	AP1	3.999	0.922	0.457	-
	AP2	4.029	0.951	0.489	-
	AP3	4.003	0.873	0.794	-
	AP4	4.089	0.840	0.844	-
Programs Offered	PO1	4.140	0.792	0.642	0.667
	PO2	4.226	0.788	0.835	0.771
	PO3	4.266	0.777	0.861	0.817
	PO4	4.069	0.873	0.759	0.781
	PO5	3.924	0.926	0.604	0.605
	PO6	3.937	1.244	0.388	-
School Facility	SF1	4.074	0.834	0.786	-
	SF2	4.264	0.805	0.794	-
	SF3	4.193	0.840	0.854	-
	SF4	4.245	0.799	0.871	-
	SF5	4.275	0.819	0.784	-
	SF6	3.926	0.914	0.719	-
	SF7	4.064	0.868	0.754	-
	SF8	4.058	0.909	0.705	-
Faculty Profile	FP1	4.202	0.769	0.653	-
	FP2	4.145	0.799	0.755	-
	FP3	3.723	0.987	0.827	-
	FP4	3.694	1.009	0.837	-
	FP5	3.819	0.906	0.796	-
Kinship Patronage	KP1	3.614	1.204	0.693	0.704
	KP2	3.328	1.321	0.637	0.640
	KP3	2.587	1.486	0.705	0.647
	KP4	3.355	1.266	0.756	0.831
University Ranking	UR1	3.824	1.067	0.439	-
	UR2	4.125	0.776	0.810	0.784
	UR3	3.910	0.839	0.739	0.769
	UR4	4.245	0.793	0.804	0.767
	UR5	3.702	0.923	0.673	0.734
University Image	UI1	3.531	1.063	0.352	-
	UI2	4.186	0.831	0.605	0.686
	UI3	3.904	0.949	0.646	0.745
Affective	A1	3.480	0.108	0.653	0.588
	A2	3.895	0.881	0.704	0.644
	A3	4.021	0.925	0.765	0.702
	A4	3.753	1.004	0.873	0.880
	A5	3.759	0.971	0.890	0.927
Autonomy	AU1	3.897	0.915	0.726	-
	AU2	3.927	0.868	0.786	-
	AU3	3.677	1.004	0.696	-
	AU4	4.351	0.790	0.551	-
	AU5	3.931	0.942	0.683	-
Relatedness	RE1	3.781	1.102	0.332	-
	RE2	4.179	0.869	0.626	0.712
	RE3	3.879	0.969	0.812	0.736
	RE4	3.699	1.027	0.825	0.790
	RE5	3.044	1.235	0.468	-
	RE6	3.132	1.280	0.328	-
	RE7	4.084	1.035	0.442	-
Competency	CO1	4.223	0.835	0.768	0.826
	CO2	4.225	0.839	0.697	0.728
	CO3	4.263	0.887	0.769	0.727
	CO4	4.251	0.851	0.856	0.863
	CO5	4.450	0.726	0.777	0.779
Empathy	EM1	3.602	1.082	0.850	0.832
	EM2	3.423	1.094	0.877	0.840
	EM3	4.484	1.132	0.755	0.762
	EM4	3.578	1.072	0.805	0.828
	EM5	3.436	1.085	0.838	0.861
Perceived Behavioral Control	PBC1	3.802	1.066	0.509	0.518
	PBC2	3.933	0.951	0.772	0.776
	PBC3	4.074	0.926	0.757	0.748
	PBC4	3.942	1.027	0.815	0.782
	PBC5	3.202	0.901	0.797	0.764
Student Satisfaction	SS1	3.890	0.945	0.793	0.800
	SS2	3.795	0.999	0.839	0.869
	SS3	3.703	1.055	0.816	0.835
	SS4	3.797	1.010	0.788	0.751
	SS5	3.708	1.061	0.848	0.831

**Table 5:** Composite reliability.

Factor	Cronbach’s α	Average Variance Extracted (AVE)	Composite Reliability (CR)
Programs Offered	0.767	0.537	0.851
Kinship Patronage	0.854	0.504	0.800
University Ranking	0.790	0.583	0.848
University Image	0.837	0.513	0.678
Affective	0.783	0.577	0.869
Relatedness	0.885	0.558	0.791
Competency	0.798	0.618	0.890
Empathy	0.881	0.681	0.914
Perceived Behavioral Control	0.941	0.560	0.862
Student Satisfaction	0.844	0.669	0.910
Student Loyalty	0.911	0.514	0.806

Table 6 presents the model fit of the study. Following the suggestion of Gefen et al. [65], the values for the Incremental Fit Index (IFI), Tucker Lewis Index (TLI), Comparative Fit Index (CFI), Goodness of Fit Index (GFI), and Adjusted Goodness of Fit Index (AGFI) should be more than 0.80. In addition, the Root Mean Square Error (RMSEA) should be less than 0.70 [66]. This study satisfies all conditions, which indicates that all the values are acceptable and have a good fit. Finally, Table 7 represents the direct, indirect, and total effects of the latent.

**Table 6:** Model fit.

Goodness of fit measures of SEM	Parameter Estimates	Minimum cut-off	Suggested by
Incremental Fit Index (IFI)	0.825	>0.80	Gefen et al. [65]
Tucker Lewis Index (TLI)	0.806	>0.80	Gefen et al. [65]
Comparative Fit Index (CFI)	0.824	>0.80	Gefen et al. [65]
Goodness of Fit Index (GFI)	0.874	>0.80	Gefen et al. [65]
Adjusted Goodness of Fit Index (AGFI)	0.840	>0.80	Gefen et al. [65]
Root Mean Square Error (RMSEA)	0.069	<0.07	Steiger [66]

**Table 7:** Direct, indirect, and total effects.

No	Variable	Direct Effect	P-Value	Indirect Effect	P-Value	Total Effect	P-Value
1	CO→PBC	0.620	0.003	-	-	0.620	0.003
2	UR→UI	0.598	0.013	-	-	0.598	0.013
3	SS→SL	0.574	0.009	-	-	0.574	0.009
4	PBC→SS	0.447	0.006	-	-	0.447	0.006
5	UI→SL	0.424	0.007	-	-	0.424	0.007
6	PO→UI	0.265	0.010	-	-	0.265	0.010
7	A→SL	0.192	0.008	-	-	0.192	0.008
8	KP→UI	0.178	0.011	-	-	0.178	0.011
9	RE→PBC	0.157	0.005	-	-	0.157	0.005
10	EM→PBC	0.124	0.012	-	-	0.124	0.012
11	CO→SS	-	-	0.230	0.009	0.230	0.009
12	UR→SL	-	-	0.204	0.003	0.204	0.003
13	PBC→SL	-	-	0.190	0.002	0.190	0.002
14	CO→SL	-	-	0.114	0.005	0.114	0.005
15	PO→SL	-	-	0.069	0.007	0.069	0.007
16	KP→SL	-	-	0.047	0.006	0.047	0.006
17	RE→SS	-	-	0.033	0.003	0.033	0.003
18	EM→SS	-	-	0.022	0.008	0.022	0.008
19	RE→SL	-	-	0.017	0.003	0.017	0.003
20	EM→SL	-	-	0.012	0.005	0.012	0.005

## 5. Discussion

This study integrated the TPB and SDT to determine factors affecting student loyalty. The researchers distributed an online survey to understand the relationship of loyalty with its antecedent variables. Consequently, Structural Equation Modelling (SEM) was utilized to determine the causal relationship among latent such as SL, UI, TF, AP, PO, SF, FP, KP, UR, SS, PBC, AU, RE, CO, EM, and AF. SEM indicated the formed direct and indirect relationship between the latent affecting overall student loyalty towards a university. Loyalty in this study is defined as the students’ pursuance and objective feeling when it comes to choosing their current institution for higher education, choosing the institution despite available choices, and talking to others to consider the current institution.

Presented in Table 8 are the summarized results for the different hypotheses created. It could be deduced that 10 out of 15 hypotheses were accepted. Several implications on why Hypotheses 1, 2, 4, 5, and 10 were insignificant. These were discussed in the following sections.

**Table 8:** Hypotheses results.

No	Relationship	Beta coefficient	p-value	Result	Significance	Hypothesis
1	TF→UI	0.053	0.114	Positive	Insignificant	Rejected
2	AP→UI	0.106	0.068	Positive	Insignificant	Rejected
3	PO→UI	0.265	0.010	Positive	Significant	Accepted
4	SF→UI	0.039	0.103	Positive	Insignificant	Rejected
5	FP→UI	0.134	0.053	Positive	Insignificant	Rejected
6	KP→UI	0.178	0.011	Positive	Significant	Accepted
7	UR→UI	0.598	0.013	Positive	Significant	Accepted
8	UI→SL	0.424	0.007	Positive	Significant	Accepted
9	A→SL	0.192	0.008	Positive	Significant	Accepted
10	AU→PBC	0.105	0.070	Positive	Insignificant	Rejected
11	RE→PBC	0.157	0.005	Positive	Significant	Accepted
12	CO→PBC	0.620	0.003	Positive	Significant	Accepted
13	EM→PBC	0.124	0.012	Positive	Significant	Accepted
14	PBC→SS	0.447	0.006	Positive	Significant	Accepted
15	SS→SL	0.574	0.009	Positive	Significant	Accepted

Results showed that CO had the highest significant direct effect on PBC (β:0.620; p = 0.003). This result may be attributed to the students’ university preferences which depend on the perceived success of the behavior. It could be interpreted that competency reflects the students’ formulated decisions in line with the future benefits of their actions. This correlation signifies that the higher the procurement of CO, the stronger the perceptions of PBC [67]. Thus, reaching a specific degree of competency will enable a student to execute behavioral control [39].

Based on the results, UR had a significant and direct effect on UI (β:0.598; p = 0.013). University performance, student-to-staff ratio, awards, citations, and general perception of the school fall under the UR indicators. In this study, students’ perception of UI is highly affected by the perceived university ranking of their school. Lukman et al. [68] mentioned that universities are extensively compared to one another based on research outputs, student-to-staff ratio, citation scores, and scientific publications. Moreover, the university’s research performance and citation scores also directly affect the UI, reflecting on the methodologies of various university-ranking publications.

The SEM indicated that SS had significant direct effects on SL (β:0.514; p = 0.009). The results showed that students’ general satisfaction with their university influences their loyalty and retention, supported by several similar studies with a positive correlation between the two variables (Amnå, 2021) [69]. Thus, this relationship implies that improving various aspects of the institution, such as facilities, equipment, and academic quality, will improve satisfaction leading to student loyalty. To further establish the result, Chandra et al. [10] stated that offering good service quality alone would not improve SL, but it should be accompanied by evaluating SS.

Moreover, PBC was seen to have a significant and direct effect on SS (β:0.447; p = 0.006). The indicators of PBC regarding the overall autonomy, control, and capability within an institution impact SS, implying that a student’s perception of how much they have control over a situation regarding their universities will directly influence their satisfaction. In addition, several studies have shown similar results, indicating that PBC has a positive correlation with satisfaction [26].

The results also indicated a significant direct influence of UI on SL (β:0.424; p = 0.007). The SEM results presented that students’ overall view of the university, the university’s level of prestige, and the recommendations of acquaintances were positively correlated with loyalty and retention towards a university. The activities accomplished by a university affect its projected image toward potential students. This relation indicates that the failure of universities to project a positive image toward students will lead to a decline in SL [70]. Furthermore, Daud et al. [71] indicated a positive influence between UI and SL, stating that activities that lead to a better impression of the university will give students higher retention levels, giving students assurance that they chose the right university.

PO and KP had significant and direct effects on UI with (β:0.265; p = 0.010) and (β:0.178; p = 0.011), respectively. The latent PO showed that students’ perceptions affected the UI as they prefer institutions with practical programs, high-quality education, and diverse opportunities. Kazoleas et al. [72] stated that institutional factors, such as the PO significantly affect UI, supporting the discussed result. Similarly, KP demonstrated the influence of alumni and parental recommendation in decision-making for institutional retention intentions, ultimately also positively affecting UI. Several studies expressed this correlation stating the parental influence in choosing universities [33].

AF had a significant and direct effect on SL (β:0.192; p = 0.008). From the results, students’ experience of belongingness to the school community contributes to the positive effect of loyalty. In addition, students who feel proud of studying at their university are likely to show loyalty by supporting the university. Therefore, students’ positive affection, such as belongingness, identification, pride, and willingness, affect loyalty towards their university. This is supported by several studies indicating that affective perceptions result in loyalty outcomes such as the recommendation to prospective students, retention intentions, and support to university endeavors [73, 74].

In addition, the SEM indicated that RE and EM had significant direct effects on PBC (β:0.157; p = 0.005) and (β:0.124; p = 0.012). The results showed that the influence, encouragement, and support of family, friends, classmates, and teachers on the student’s decision affect students’ PBC. The connection and sense of belongingness of the student to the institution also influence the student’s PBC. Park et al. [75] showed that relatedness positively affects the PBC as relatedness affects a person’s decision. Similarly, EM indicates the school’s concern towards students’ individual needs regarding inquiries, organization, and convenience to the welfare of students. The indicators such as influence, encouragement, and support of family, friends, classmates, and teachers signify that EM has an impact on PBC. De Leeuw et al. [23] supported this result, stating that universities with higher empathy responded positively to all TPB measures, including PBC.

Regarding the indirect correlations, CO had the highest indirect effect on SS (β:0.230; p = 0.009). Under SDT, CO is essential in forming an individual’s satisfaction [76]. Similarly, Teixeira et al. [77] incorporated SDT with TPB and found the mediating role of PBC within the relationship between CO and SS. Moreover, Sopiah et al. [78] stated that CO has a significant indirect effect on SS. Therefore, CO indirectly affects satisfaction through PBC as a mediator. Meanwhile, SEM indicated that UR had significant indirect effects on SL (β:0.204, p = 0.003). Hasan and Hosen [79] mentioned external prestige, specifically UR SL. This study shows that the student’s perception of UR influences SL through UI as the mediator.

From the results, PBC (β:0.190; p = 0.002) and CO had significant indirect effects on SL (β:0.114; p = 0.005). Nguyen and Khoa [80] support the indirect effect of perceived PBC in SL, suggesting the presence of a mediating variable. Thus, in this study, PBC and CO indirectly influence SL through satisfaction as the mediator.

### 5.1 Theoretical contribution

Overall, the formed framework of this study indicates a significant relationship between the latent constructs, which culminates in the formation of SL. The integration of SDT and TPB in this study revealed the influence of their various constructs on SL. As seen from the results, CO under the SDT had the highest direct relationship affecting PBC under the TPB. This indicates that students’ perception of their ability is the most significant factor that affects the intention of the behaviour among other factors. This correlation extends itself in the linear progression of competency towards SS and SL. The integration of SDT and PBC can be utilized to determine the intention of a person to perform a specific action. Thus, this integrated theoretical framework can be applied by different universities as a reference to measure SS, UI, and SL.

### 5.2 Practical implications

There are various ways to improve student retention within an academic institution. Considering the results, academic institutions should improve upon the three antecedents of SL, which are UI, AF, and SS. This is because the three antecedents have proven to have a high significant direct effect on student loyalty. Developing and improving these three require considering their antecedents as well. For example, UR was one of the most significant indicators of UI, meaning having a prestigious university will lead to a better external image and therefore lead to student retention. In addition, universities should also maintain their relations with alumni to project a positive perception of the university to potential students. Creating programs that fit the interests of students is also beneficial for improving UI.

Developing a welcoming atmosphere within the university will significantly impact the student belongingness, therefore improving the affective aspect. For example, teachers who deliver their lectures while consistently engaging with the students and assessing their learning will improve the affective aspect. Having approachable university members (university directors and employees) will also give students a sense of belongingness. When ensuring satisfaction, universities may consider encouraging autonomy among students and learning to connect to students through relatedness and empathy. Considering students as customers, the satisfaction of students would result in herd application. The increase in the application would result in an increase in population, sales, marketability, and profitability of the university.

### 5.3 Limitation

Considering the setup of the current study, several limitations were considered. First, this study focused on senior high school students going to college to pursue higher education. It is recommended to quantify the results of this study with students already in college or higher education. The reason for their choice could result in additional information to promote student retention. Second, the study was conducted during the COVID-19 pandemic. Thus, the subjective measurement of the participants was based on their experiences with the online class setup [81, 82]. Considering the students’ behavior, the current situation influences students’ stress and attitudes toward the new normal education. Therefore, this study can be conducted when the education setup is normalized into face-to-face classes or blended learning. In addition, the consideration of other factors and variables may provide more insights. Since this study adopted several variables from established theories and tried the integration, factors such as motivational aspects may be considered. Finally, this study only considered the students’ perceptions and not their actual experiences in the university. Their preferences may also be considered as an extension of this study to further elaborate on what factors significantly affect the intention of students to stay in the university for their higher education and accurately measure satisfaction and loyalty.

## 6. Conclusion

SL generally refers to the formed bond between the student and an institution. This relationship between a university and its students proves essential in a university’s success in the competitive field of higher education institutions. The loyalty of students to their university may result in positive word-of-mouth to prospective students, an increase in student retention rates, and support in university endeavors.

The aim of this study was to determine students’ loyalty among high school students to pursue their college or higher education in their current universities by utilizing SDT and Extended TPB. The results showed that CO had the highest direct significant effect on PBC, leading to student loyalty in the same institution, followed by UR, and SS. Furthermore, mediators were identified in this study. First, PBC was found to mediate the effects of CO, RE, and EM on SS. This showed that students’ cognitive ability is the primary reason to choose a university, in line with their self-awareness. Second, UI mediates the effects of UR, PO, and KP on SL. Lastly, SS acts as a mediator between PBC and SL. This showed that having a prestigious university will lead to a better external image and therefore lead to student retention. To which, 10 out of 15 hypotheses were accepted. In addition, universities should also maintain their relations with alumni to project a positive perception of the university to potential students.

The results of this study could be applied in focusing university efforts on specific aspects of university image and satisfaction in targeting student loyalty. Schools may improve the competency of their students through career and college orientations. Thus, an improvement in student competency may result in the development of PBC, SS, and overall loyalty as supported by the result of this study. In addition, universities may focus on enhancing school performance and ranking in the competitive field of higher education institutions. The improvement in UR will directly affect UI and SL.

## Supporting information

S1 Appendix (DOCX)
