# Supplementary material for: Determination of loyalty among high school students to retain in the same university for higher education: An integration of Self-Determination Theory and Extended Theory of Planned Behavior
Source: PLoS One. 2023 Nov 8;18(11):e0286185. doi: 10.1371/journal.pone.0286185 (PMC10631657; doi:10.1371/journal.pone.0286185)
Supplement: S1 Appendix — (DOCX) [file pone.0286185.s001.docx]

**Appendix A.** Questionnaire

Greetings! This is an online survey for Senior High School Students to measure their loyalty to retain in the same university for higher education (undergraduate degree). The measurement will be measured through a 5-point Likert Scale (5-Strongly Agree, 1-Strongly Disagree). Your participation in this research is voluntary. If you choose not to participate in this study or decide to withdraw the survey, you may do so at any time without being penalized. If you do decide on partaking in this research, your participation will be greatly appreciated. You will receive no direct benefits from participating in this research; however, your responses may be substantial data that will aid in achieving the objectives of this research. All of the information that will be collected will be of essence to the research. Consequently, they will all be held confidential. They will be also be used solely for scholarly purposes, and may be shared with Mapúa University representatives. All data will be stored in a password protected electronic format. Please click the agree button to proceed with the survey.

Section 1: Demographics

Gender ᵒ Male ᵒ Female ᵒ Other

Age ᵒ 14 ᵒ 15 ᵒ 16 ᵒ 17 ᵒ 18 ᵒ 19 ᵒ 20

Grade Level ᵒ Grade 9 ᵒ Grade 10 ᵒ Grade 11 ᵒ Grade 12

Salary of Parents ᵒ Less than 15,000 ᵒ 15,000-30,000 ᵒ 30,001-45,000

ᵒ 45,001-60,000 ᵒ 60,001-75,000 ᵒ Greater than 75,000

Section 2: This section covers several indicators to measure factors affecting students’ retention in the same university for higher education (college). Please answer using the 5-point Likert Scale as presented wherein 5 – Strongly Agree and 1 – Strongly Disagree will be the scale. A total of 80 questions are available and answering the survey will take 15-20 minutes. We would like to thank you for your participation in this study.

| I intend to continue my college studies in the same institution. | | | | |
| --- | --- | --- | --- | --- |
| ᵒ | ᵒ | ᵒ | ᵒ | ᵒ |
| Strongly Disagree |  |  |  | Strongly Agree |
| I intend to continue my college studies in the same institution. | | | | |
| ᵒ | ᵒ | ᵒ | ᵒ | ᵒ |
| Strongly Disagree |  |  |  | Strongly Agree |
| I would choose my present institution if I had to choose a school for college right now. | | | | |
| ᵒ | ᵒ | ᵒ | ᵒ | ᵒ |
| Strongly Disagree |  |  |  | Strongly Agree |
| I often talk to my peers about the benefits of joining my current school. | | | | |
| ᵒ | ᵒ | ᵒ | ᵒ | ᵒ |
| Strongly Disagree |  |  |  | Strongly Agree |
| My family's financial capacity plays a significant role in choosing my school for college. | | | | |
| ᵒ | ᵒ | ᵒ | ᵒ | ᵒ |
| Strongly Disagree |  |  |  | Strongly Agree |
| I consider tuitions fee when choosing schools. | | | | |
| ᵒ | ᵒ | ᵒ | ᵒ | ᵒ |
| Strongly Disagree |  |  |  | Strongly Agree |
| I consider schools that offer financial discounts. | | | | |
| ᵒ | ᵒ | ᵒ | ᵒ | ᵒ |
| Strongly Disagree |  |  |  | Strongly Agree |
| The tuition fee in my choice is reasonable. | | | | |
| ᵒ | ᵒ | ᵒ | ᵒ | ᵒ |
| Strongly Disagree |  |  |  | Strongly Agree |
| I consider the admission process of the school/s that I choose. | | | | |
| ᵒ | ᵒ | ᵒ | ᵒ | ᵒ |
| Strongly Disagree |  |  |  | Strongly Agree |
| I consider schools that offer/s online admission. | | | | |
| ᵒ | ᵒ | ᵒ | ᵒ | ᵒ |
| Strongly Disagree |  |  |  | Strongly Agree |
| The admission process of my current school is easy to understand. | | | | |
| ᵒ | ᵒ | ᵒ | ᵒ | ᵒ |
| Strongly Disagree |  |  |  | Strongly Agree |
| My current school has reasonable admission requirements. | | | | |
| ᵒ | ᵒ | ᵒ | ᵒ | ᵒ |
| Strongly Disagree |  |  |  | Strongly Agree |
| The programs of the institution offer practical and useful content that can be utilized for future use. | | | | |
| ᵒ | ᵒ | ᵒ | ᵒ | ᵒ |
| Strongly Disagree |  |  |  | Strongly Agree |
| The college programs in my current school offer high-quality academic standards. | | | | |
| ᵒ | ᵒ | ᵒ | ᵒ | ᵒ |
| Strongly Disagree |  |  |  | Strongly Agree |
| My institution offers programs with high quality education. | | | | |
| ᵒ | ᵒ | ᵒ | ᵒ | ᵒ |
| Strongly Disagree |  |  |  | Strongly Agree |
| The programs offered by my current institution ensure student development. | | | | |
| ᵒ | ᵒ | ᵒ | ᵒ | ᵒ |
| Strongly Disagree |  |  |  | Strongly Agree |
| My institution offers various courses to fit every student. | | | | |
| ᵒ | ᵒ | ᵒ | ᵒ | ᵒ |
| Strongly Disagree |  |  |  | Strongly Agree |
| My current institution offers the course that I want to take. | | | | |
| ᵒ | ᵒ | ᵒ | ᵒ | ᵒ |
| Strongly Disagree |  |  |  | Strongly Agree |
| My school has overall quality facilities. | | | | |
| ᵒ | ᵒ | ᵒ | ᵒ | ᵒ |
| Strongly Disagree |  |  |  | Strongly Agree |
| My school offers advanced technology or special learning equipment in their facilities. | | | | |
| ᵒ | ᵒ | ᵒ | ᵒ | ᵒ |
| Strongly Disagree |  |  |  | Strongly Agree |
| My school is equipped with sufficient laboratories. | | | | |
| ᵒ | ᵒ | ᵒ | ᵒ | ᵒ |
| Strongly Disagree |  |  |  | Strongly Agree |
| My school has adequate facilities for learning. | | | | |
| ᵒ | ᵒ | ᵒ | ᵒ | ᵒ |
| Strongly Disagree |  |  |  | Strongly Agree |
| My school has sufficient on-site service facility such as clinics, school canteen, and counselling center. | | | | |
| ᵒ | ᵒ | ᵒ | ᵒ | ᵒ |
| Strongly Disagree |  |  |  | Strongly Agree |
| My school has adequate recreational facilities. | | | | |
| ᵒ | ᵒ | ᵒ | ᵒ | ᵒ |
| Strongly Disagree |  |  |  | Strongly Agree |
| My school has good classroom conditions. | | | | |
| ᵒ | ᵒ | ᵒ | ᵒ | ᵒ |
| Strongly Disagree |  |  |  | Strongly Agree |
| My school offers clean and well-maintained comfort rooms. | | | | |
| ᵒ | ᵒ | ᵒ | ᵒ | ᵒ |
| Strongly Disagree |  |  |  | Strongly Agree |
| Professors are up to date with information regarding their field of profession. | | | | |
| ᵒ | ᵒ | ᵒ | ᵒ | ᵒ |
| Strongly Disagree |  |  |  | Strongly Agree |
| The instructors are highly qualified in their field of teaching. | | | | |
| ᵒ | ᵒ | ᵒ | ᵒ | ᵒ |
| Strongly Disagree |  |  |  | Strongly Agree |
| The instructors give individual attention to each student. | | | | |
| ᵒ | ᵒ | ᵒ | ᵒ | ᵒ |
| Strongly Disagree |  |  |  | Strongly Agree |
| The instructors understand what each student needs. | | | | |
| ᵒ | ᵒ | ᵒ | ᵒ | ᵒ |
| Strongly Disagree |  |  |  | Strongly Agree |
| I am satisfied with how the instructors deliver lectures. | | | | |
| ᵒ | ᵒ | ᵒ | ᵒ | ᵒ |
| Strongly Disagree |  |  |  | Strongly Agree |
| My alumni relatives highly recommend my current institution. | | | | |
| ᵒ | ᵒ | ᵒ | ᵒ | ᵒ |
| Strongly Disagree |  |  |  | Strongly Agree |
| My alumni relatives significantly affect my choice of school. | | | | |
| ᵒ | ᵒ | ᵒ | ᵒ | ᵒ |
| Strongly Disagree |  |  |  | Strongly Agree |
| I chose my current institution because of my relatives are alumni of the school. | | | | |
| ᵒ | ᵒ | ᵒ | ᵒ | ᵒ |
| Strongly Disagree |  |  |  | Strongly Agree |
| The alumni of my school highly recommend the institution. | | | | |
| ᵒ | ᵒ | ᵒ | ᵒ | ᵒ |
| Strongly Disagree |  |  |  | Strongly Agree |
| I consider institutional ranking when choosing a school. | | | | |
| ᵒ | ᵒ | ᵒ | ᵒ | ᵒ |
| Strongly Disagree |  |  |  | Strongly Agree |
| My school has a great Institutional Ranking. | | | | |
| ᵒ | ᵒ | ᵒ | ᵒ | ᵒ |
| Strongly Disagree |  |  |  | Strongly Agree |
| My school has a considerable student to academic staff ratio. | | | | |
| ᵒ | ᵒ | ᵒ | ᵒ | ᵒ |
| Strongly Disagree |  |  |  | Strongly Agree |
| My school has a high academic reputation such as awards and highly cited researchers. | | | | |
| ᵒ | ᵒ | ᵒ | ᵒ | ᵒ |
| Strongly Disagree |  |  |  | Strongly Agree |
| I feel that my school is greater than other schools. | | | | |
| ᵒ | ᵒ | ᵒ | ᵒ | ᵒ |
| Strongly Disagree |  |  |  | Strongly Agree |
| I choose schools based on their level of prestige. | | | | |
| ᵒ | ᵒ | ᵒ | ᵒ | ᵒ |
| Strongly Disagree |  |  |  | Strongly Agree |
| I have heard that the graduates of my current institution have become successful. | | | | |
| ᵒ | ᵒ | ᵒ | ᵒ | ᵒ |
| Strongly Disagree |  |  |  | Strongly Agree |
| I have always had a good impression of my current school. | | | | |
| ᵒ | ᵒ | ᵒ | ᵒ | ᵒ |
| Strongly Disagree |  |  |  | Strongly Agree |
| I think my current institution is the best choice among my other school choices. | | | | |
| ᵒ | ᵒ | ᵒ | ᵒ | ᵒ |
| Strongly Disagree |  |  |  | Strongly Agree |
| I am willing to make all efforts in helping my current school achieve its goals. | | | | |
| ᵒ | ᵒ | ᵒ | ᵒ | ᵒ |
| Strongly Disagree |  |  |  | Strongly Agree |
| I feel proud to study in my current institution. | | | | |
| ᵒ | ᵒ | ᵒ | ᵒ | ᵒ |
| Strongly Disagree |  |  |  | Strongly Agree |
| I feel a strong sense of belonging in my current school. | | | | |
| ᵒ | ᵒ | ᵒ | ᵒ | ᵒ |
| Strongly Disagree |  |  |  | Strongly Agree |
| I feel a strong sense of identification with my current school. | | | | |
| ᵒ | ᵒ | ᵒ | ᵒ | ᵒ |
| Strongly Disagree |  |  |  | Strongly Agree |
| My current campus provides activities that enable us to use our own set of skills. | | | | |
| ᵒ | ᵒ | ᵒ | ᵒ | ᵒ |
| Strongly Disagree |  |  |  | Strongly Agree |
| The teachers in my current school provide me with choices and options. | | | | |
| ᵒ | ᵒ | ᵒ | ᵒ | ᵒ |
| Strongly Disagree |  |  |  | Strongly Agree |
| I do not feel restricted when I'm at my current school. | | | | |
| ᵒ | ᵒ | ᵒ | ᵒ | ᵒ |
| Strongly Disagree |  |  |  | Strongly Agree |
| Our professors encourage us to ask questions. | | | | |
| ᵒ | ᵒ | ᵒ | ᵒ | ᵒ |
| Strongly Disagree |  |  |  | Strongly Agree |
| I feel that I can decide on my own freely. | | | | |
| ᵒ | ᵒ | ᵒ | ᵒ | ᵒ |
| Strongly Disagree |  |  |  | Strongly Agree |
| My relationship with my family, friends, classmates, and teachers influences my decision when choosing schools. | | | | |
| ᵒ | ᵒ | ᵒ | ᵒ | ᵒ |
| Strongly Disagree |  |  |  | Strongly Agree |
| I feel the support of my family, friends, classmates, and teachers when choosing a school. | | | | |
| ᵒ | ᵒ | ᵒ | ᵒ | ᵒ |
| Strongly Disagree |  |  |  | Strongly Agree |
| I feel the social support of the people in my current school. | | | | |
| ᵒ | ᵒ | ᵒ | ᵒ | ᵒ |
| Strongly Disagree |  |  |  | Strongly Agree |
| I feel connected and part of my current school. | | | | |
| ᵒ | ᵒ | ᵒ | ᵒ | ᵒ |
| Strongly Disagree |  |  |  | Strongly Agree |
| I feel that my friends will go to the same institution as me. | | | | |
| ᵒ | ᵒ | ᵒ | ᵒ | ᵒ |
| Strongly Disagree |  |  |  | Strongly Agree |
| My friends encourage me to enroll in the institution where they are at. | | | | |
| ᵒ | ᵒ | ᵒ | ᵒ | ᵒ |
| Strongly Disagree |  |  |  | Strongly Agree |
| My parents/relatives encourage me to enroll in the institution of my choice. | | | | |
| ᵒ | ᵒ | ᵒ | ᵒ | ᵒ |
| Strongly Disagree |  |  |  | Strongly Agree |
| I can set my preferences in choosing a school. | | | | |
| ᵒ | ᵒ | ᵒ | ᵒ | ᵒ |
| Strongly Disagree |  |  |  | Strongly Agree |
| I apply my past and current experiences to set my preferences in choosing a school. | | | | |
| ᵒ | ᵒ | ᵒ | ᵒ | ᵒ |
| Strongly Disagree |  |  |  | Strongly Agree |
| I can distinguish between the schools I want, and I do not want. | | | | |
| ᵒ | ᵒ | ᵒ | ᵒ | ᵒ |
| Strongly Disagree |  |  |  | Strongly Agree |
| I can select the school I want based on my preference. | | | | |
| ᵒ | ᵒ | ᵒ | ᵒ | ᵒ |
| Strongly Disagree |  |  |  | Strongly Agree |
| Overall, I believed that choosing a school based on my preferences will be beneficial to me. | | | | |
| ᵒ | ᵒ | ᵒ | ᵒ | ᵒ |
| Strongly Disagree |  |  |  | Strongly Agree |
| My school makes an effort to understand students' needs. | | | | |
| ᵒ | ᵒ | ᵒ | ᵒ | ᵒ |
| Strongly Disagree |  |  |  | Strongly Agree |
| My current school knows the needs of its students. | | | | |
| ᵒ | ᵒ | ᵒ | ᵒ | ᵒ |
| Strongly Disagree |  |  |  | Strongly Agree |
| I believe that my current school has reasonable schedules convenient to its students. | | | | |
| ᵒ | ᵒ | ᵒ | ᵒ | ᵒ |
| Strongly Disagree |  |  |  | Strongly Agree |
| My school actively responds to students' enquiries. | | | | |
| ᵒ | ᵒ | ᵒ | ᵒ | ᵒ |
| Strongly Disagree |  |  |  | Strongly Agree |
| My school gives students individual attention to its students. | | | | |
| ᵒ | ᵒ | ᵒ | ᵒ | ᵒ |
| Strongly Disagree |  |  |  | Strongly Agree |
| I am confident that I could stay in the same school if I want to. | | | | |
| ᵒ | ᵒ | ᵒ | ᵒ | ᵒ |
| Strongly Disagree |  |  |  | Strongly Agree |
| I have the knowledge and control where to enroll. | | | | |
| ᵒ | ᵒ | ᵒ | ᵒ | ᵒ |
| Strongly Disagree |  |  |  | Strongly Agree |
| I think that I am capable of choosing a school for college on my own. | | | | |
| ᵒ | ᵒ | ᵒ | ᵒ | ᵒ |
| Strongly Disagree |  |  |  | Strongly Agree |
| I have the overall control in choosing a school for college. | | | | |
| ᵒ | ᵒ | ᵒ | ᵒ | ᵒ |
| Strongly Disagree |  |  |  | Strongly Agree |
| I will choose the institution I will be going to. | | | | |
| ᵒ | ᵒ | ᵒ | ᵒ | ᵒ |
| Strongly Disagree |  |  |  | Strongly Agree |
| In general, I am satisfied with the services offered by my current school. | | | | |
| ᵒ | ᵒ | ᵒ | ᵒ | ᵒ |
| Strongly Disagree |  |  |  | Strongly Agree |
| I am pleased with my decision to enroll on this campus. | | | | |
| ᵒ | ᵒ | ᵒ | ᵒ | ᵒ |
| Strongly Disagree |  |  |  | Strongly Agree |
| I am satisfied to study on the same institution I am currently at. | | | | |
| ᵒ | ᵒ | ᵒ | ᵒ | ᵒ |
| Strongly Disagree |  |  |  | Strongly Agree |
| My experience in my school has been enjoyable. | | | | |
| ᵒ | ᵒ | ᵒ | ᵒ | ᵒ |
| Strongly Disagree |  |  |  | Strongly Agree |
| I am enjoying studying at my current school. | | | | |
| ᵒ | ᵒ | ᵒ | ᵒ | ᵒ |
| Strongly Disagree |  |  |  | Strongly Agree |
